# Memory effect and magnetocrystalline anisotropy impact on the surface magnetic domains of magnetite(001)

**DOI:** 10.1038/s41598-018-24160-1

**Published:** 2018-04-16

**Authors:** Laura Martín-García, Gong Chen, Yaiza Montaña, Arantzazu Mascaraque, Beatriz M. Pabón, Andreas K. Schmid, Juan de la Figuera

**Affiliations:** 1 Instituto de Química Física “Rocasolano”, CSIC, Madrid, E-28006 Spain; 2 NCEM, Molecular Foundry, Lawrence Berkeley National Laboratory, Berkeley, California 94720 USA; 3 Dpto. de Física de Materiales, Universidad Complutense de Madrid, Madrid, E-28040 Spain; 4 Unidad Asociada IQFR(CSIC)-UCM, Madrid, E-28040 Spain

## Abstract

The structure of magnetic domains, i.e. regions of uniform magnetization separated by domain walls, depends on the balance of competing interactions present in ferromagnetic (or ferrimagnetic) materials. When these interactions change then domain configurations also change as a result. Magnetite provides a good test bench to study these effects, as its magnetocrystalline anisotropy varies significantly with temperature. Using spin-polarized electron microscopy to map the micromagnetic domain structure in the (001) surface of a macroscopic magnetite crystal (~1 cm size) shows complex domain patterns with characteristic length-scales in the micrometer range and highly temperature dependent domain geometries. Although heating above the Curie temperature erases the domain patterns completely, cooling down reproduces domain patterns not only in terms of general characteristics: instead, complex microscopic domain geometries are reproduced in almost perfect fidelity between heating cycles. A possible explanation of the origin of the high-fidelity reproducibility is suggested to be a combination of the presence of hematite inclusions that lock bulk domains, together with the strong effect of the first order magnetocrystalline anisotropy which competes with the shape anisotropy to give rise to the observed complex patterns.

## Introduction

Cycling a macroscopic crystal of magnetic material between above and below its Curie temperature (Tc) usually results in the formation of magnetic domain patterns in the low temperature phase^1,2^. In some cases, such as when the characteristic length scale of domain sizes is similar to sample size, the domain patterns in the low temperature phase approach energy minimum configurations; one example is the stability of Landau closure domains in small magnetic particles. In such cases the observed geometry of domains may be reproduced in detail after each heating/cooling cycle. More commonly though, in macroscopic samples in which domains are small compared to sample dimensions, only the general properties of domain patterns - such as average domain sizes, domains magnetized along common easy axes, and common domain wall orientations - are reproduced after temperature cycles. But usually, the detailed geometry of a sample’s magnetic domain structure is – ‘erased’ each time the sample is heated above Tc, and domain patterns in the low temperature part of the cycle only share general resemblance but not detailed geometry. Magnetite, arguably the oldest known magnetic material^3^, is not expected to behave differently. It is a soft magnet^2^, as at room temperature the magnetocrystalline anisotropy is described to first order by *K*_1_ = −1.25·10^4^ Jm^−3^. As the negative value of *K*_1_ indicates, the magnetic bulk easy axes are along 〈111〉 directions. That is the case not only for room temperature, but for the temperature range that spans from the spin-reorientation transition temperature T_*srt*_ (also called the isotropic point, ~130 K) up to the Curie temperature. At T_*srt*_, the first order magnetocrystalline anisotropy *K*_1_ changes sign, corresponding to a change in the magnetic easy axes^4^. The evolution of the magnetocrystalline anisotropy in the cubic phase of magnetite is non-monotonic^4–7^: while it decreases with decreasing temperature from the Curie point to 210 K, it increases with decreasing temperature down to T_*srt*_.

The characterization of the magnetic domains at the magnetite surface has been mostly performed on the most compact surfaces: the (111), (100) and (110) orientations. The (110) surface comprises magnetic in-plane easy-axes, and thus, it presents closure domains that resemble bulk domains^8^. In contrast, both the (111) and the (100) lack easy-axes within the surface plane. Thereby they correspond to the case of strongly-misoriented magnetic surfaces, where the competition between anisotropies is expected to give rise to complex patterns^2^. In fact, both surfaces present a multiscale complex structure of magnetic domains first detected by Kerr microscopy on (111) surfaces^9^ and by photoemission electron microscopy on the (100) surface^10^.

There are few experiments following the evolution of the domains of magnetite with temperature. Transmission electron microscopy^11^ has provided a direct view of the magnetic and structural changes of micron-sized crystals through the Verwey transition^12,13^. High temperature domain observations were first performed by the Bitter technique on magnetite grains revealing a strong temperature dependence of the domain structure^14^. However, this technique is only suitable for experiments where the temperature remains below 620 K because of the instability of the ferrofluid at higher temperatures. This limitation can be overcome by employing Kerr microscopy. A. Ambatiello *et al*.^9^ studied the evolution of the magnetic domains on magnetite (110) at temperatures up to close to the Curie temperature, showing in this case no significant changes in the shape and type of domains but a somehow reversible domain structure after heating and cooling cycles and a significant increase in the mean domain width for temperatures between 670 K and the Curie point.

In a previous work^15^ we imaged at cryogenic temperatures the evolution of the magnetic domains on magnetite (001), when crossing the spin reorientation transition and the Verwey transition. Using a variable temperature spin-polarized low-energy electron microscope (SPLEEM)^16^ we detected that the spin reorientation transition takes place in two stages with a discontinuous change of magnetization followed by a continuous one. In this work we focus on the evolution and fate of the magnetic domains on magnetite (001) at high temperatures, from the T_*srt*_ up to the Curie point.

## Results and Discussion

**Figure 1:**
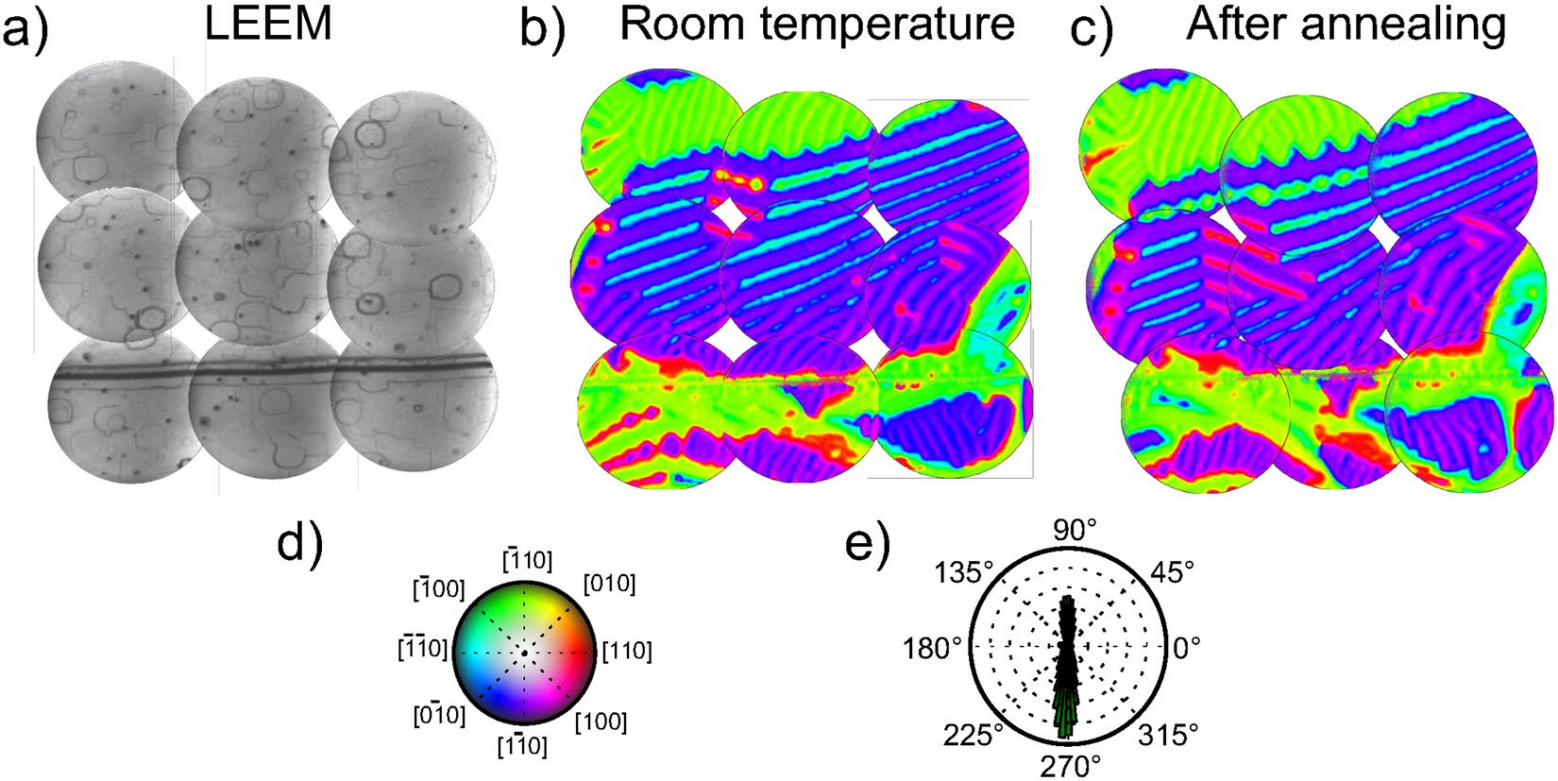
(**a**) Mosaic of 9 LEEM images that cover an area of about 30 × 30 *μ*m^2^ (each individual image has a field of view of 12 *μ*m). The x-axis and y-axis directions correspond to [110] and $[\bar{1}\mathrm{10]}$ respectively. (**b**) Vector magnetization map obtained at room temperature combining SPLEEM images acquired along three orthogonal axes in the same area shown in (**a**). (**c**) Vector magnetization map of the same area acquired after annealing the crystal for several hours at 873 K and cooling back to room temperature. (**d**) Colourmap with hue colour wheel indicating the azimuthal angle and brightness indicating the polar angle. (**e**) Histogram of the in-plane component of the magnetization from (**b**).

We have previously described the room temperature surface morphology and magnetic domain patterns on magnetite (001)^15,17^. Here we show in Fig. 1a a mosaic of LEEM images to circumvent the field-of-view limitations of the SPLEEM instrument, and highlight the more relevant features. The LEEM images show the surface covered with squarish protrusions. Those are common in magnetite (001) samples which have been cleaned by sputtering and annealing cycles^18^, and their sides correspond to the compact directions for steps in such a surface. We note that the lines in the LEEM image are most likely step bunches, although the fainter ones might correspond to atomic steps. In addition to the “mesas” a scratch is observed in the lower side crossing the area under observation.

As described in the experimental section, the magnetization vector can be measured by combining SPLEEM asymmetry images along different directions. For the same region imaged in LEEM, the magnetization vector distribution is shown in Fig. 1b. The magnetization is within the plane. The mosaic presents a complex magnetic domain distribution. A central area shows a mostly purple region (which corresponds to the magnetization being oriented along $\mathrm{[1}\bar{1}\mathrm{0]}$) with red ([110]) and cyan stripes ($[\bar{1}\bar{1}\mathrm{0]}$). Where the stripes become wider, they are seen to be surrounding green ($[\bar{1}\mathrm{10]}$) areas, sometimes with a square shape. Around this central region, extended green areas are present. In particular in the upper part, this extended region shows yellow bands with a periodicity of one micron which corresponds to a zig-zag in-plane canting of up to 30°. We have observed such domain patterns, previously reported by PEEM^10^, on different magnetite crystals, both synthetic^19^ and natural^17,20^.

The magnetic easy axes at room temperature are the 〈111〉 ones, as indicated by the negative first order cubic anisotropy constant *K*_1_. But that is only true for a bulk environment. Near the (001) surface the shape anisotropy competes with the magnetocrystalline anisotropy, with the former pushing the magnetization into the surface plane. If the shape anisotropy is much larger than the magnetocrystalline one, we should expect the magnetization to be completely in the (001) plane. And within the (001) plane it should lie along the 〈110〉 directions, driven by the residual magnetocrystalline anisotropy as the shape anisotropy does not provide any in-plane preference. A way to rapidly identify the easy axes all over the area under observation is to plot an histogram of the distribution of magnetization azimuthal angles, as shown in Fig. 1e. It is clear that the magnetization is oriented mostly either along $\mathrm{[1}\bar{1}\mathrm{0]}$ or $[\bar{1}\mathrm{10]}$ directions, which correspond to the purple and green areas in the colour image, as expected. But in addition to the average magnetization direction, the SPLEEM data show more detail. Besides the additional structure within each large purple or green domain, the magnetization map in Fig. 1b shows that the boundaries between the green and purple domains are composed of stripes which are either red or cyan. In another words, the 180° domain walls show an in-plane magnetization perpendicular to the adjacent domains, i.e. they are Néel caps with no preferred chirality^17^.

The sample was annealed up to 873 K in steps of ~100 K stopping for about one hour at each temperature in order to study the evolution of the magnetic domains with temperature. But before discussing the step-by-step evolution, we present in Fig. 1c the domains after the sample was cooled down back to room temperature. It is striking how similar the overall domain distribution is to the configuration before annealing. Ambatiello *et al*.^9^ reported, too, a strong reproducibility of the domain structure on magnetite (110) in their Kerr microscopy observations. But in our case, details can be resolved down to the nanometer scale. The same purple domain covers most of the central area under observation, with green domains around it and the domain walls between the two regions have mostly the same sense. Very similar yellow bands are observed in the green domains, which are surrounded by wavy cyan domain walls. In fact, only some details have changed: we note some modifications in the stripes within the purple domain, and within the wavy bands in the green upper domain. In the purple domain, the distribution of stripes has changed, with more red stripes along the central area. And the domain wall around a purple domain in the lower right corner, was initially split into two different orientations, red and cyan, while after the annealing it has only one orientation, cyan.

**Figure 2:**
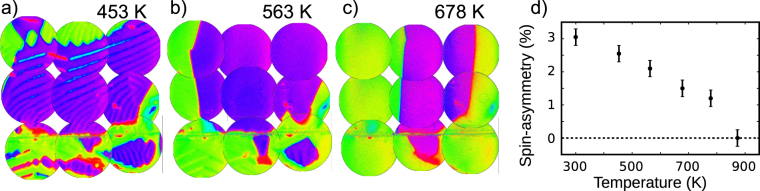
Evolution with temperature of the magnetic domains on magnetite. The same mosaic of images is shown for 453 K (**a**), 563 K (**b**) and 678 K (**c**). The colourmap is the same as in the previous figure. (**d**) Plot of the experimental spin-asymmetry versus temperature.

The evolution with temperature is shown in Fig. 2, where the same area is imaged at 453, 563 and 678 K respectively. The main difference in the first image (453 K) is that the stripes within the purple domain and the wavy bands within the green ones are fainter. In the next frame, all the microstructure within the purple domain has disappeared, while some stripes are still observed in the lower left corner on the green domain. The domain walls are becoming more straight, and Néel caps are still observed along them. At 678 K each large magnetic domain presents a uniform orientation. From then on to the Curie temperature only a gradual decrease of the magnetic contrast is observed together with some motion of the 180° domain walls. The sample was heated up to a final temperature of 873 K, and held at this temperature for several hours. During this time, a full mosaic of SPLEEM images was measured again. In none of them any contrast could be observed. The spin-asymmetry, i.e., the image contrast of the SPLEEM images, should be a good proxy of the magnetization^21^. We have plotted the evolution of the experimental spin-asymmetry with temperature in Fig. 2d (the actual spin-asymmetry is higher as the spin-polarization of the electron beam is lower than 100%). Finally, as discussed before, the sample was cooled down to room temperature where the domain distribution was recorded again (Fig. 1c).

Then the sample was cooled to room temperature, and the domain distribution was recorded again. As described before, the domain pattern after this process is very similar to the original one, as shown in Fig. 1c. The two obvious questions posed by these observations, are: (i) why do the domains change so much with temperature even when the sample is still far from the Curie temperature? And (ii) what is the origin of the strong reproducibility of the magnetic domains before and after the annealing cycle?

**Figure 3:**
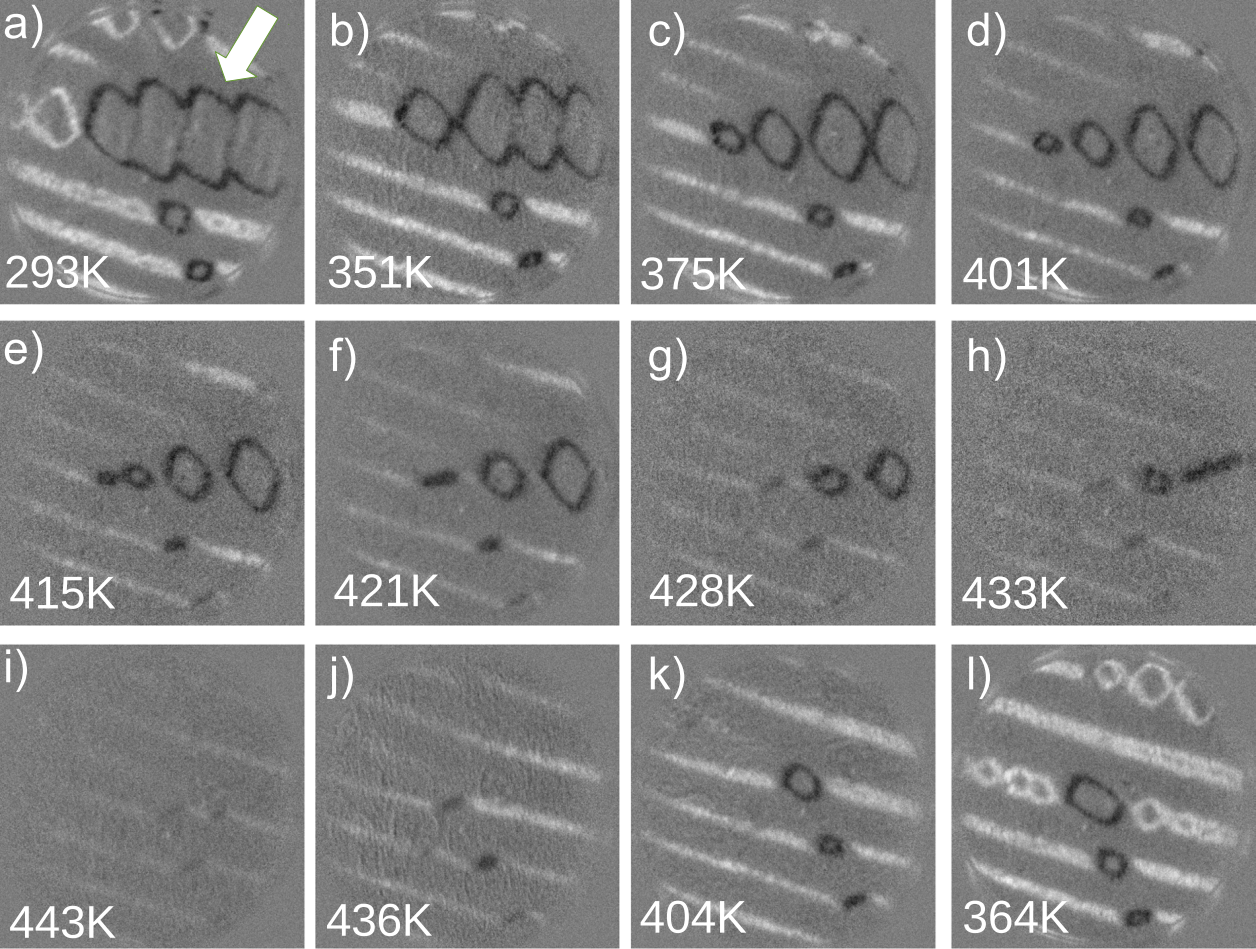
(**a**–**l**) SPLEEM asymmetry images showing the magnetization component along the x-axis, selected from a sequence of images saved during the annealing process. The crystal was heated from room temperature to 443 K (**a**–**i**), and cooled back to 364 K (**i**–**l**). The field of view is 12 *μ*m.

To answer the first question we explore the effect of temperature changes closer to room temperature. The evolution shown on a larger scale in Fig. 2 was followed in detail by acquiring movie sequences at the upper left corner of the mosaic images of Fig. 1 while the temperature was varied continuously, with the electron beam spin polarization fixed along the [110] direction (Fig. 3). Thus, the images are white when there is a component of the magnetization along the [110] direction, black when the component of the magnetization is along $[\bar{1}\bar{1}\mathrm{0]}$, and grey when the component along the x-axis is zero. It is clear that as the temperature is increased, there are different stages. First, the domain at the center which presents bands (marked with an arrow) shrinks in size and breaks up into smaller rombohedral domains, between 293 K and 375 K. Then those rombohedral domains decrease in size until they collapse into lines (401–433 K), and eventually the lines decrease in contrast to nearly disappear at 443 K. The reversibility of the process is shown by cooling from 443 K down to 364 K: the stripes first reappear, and although the square domains also reappear, they do it in a somewhat different distribution than during the heating ramp.

What is then the origin of the significant changes of the magnetic domain micro-structure as a result of changing the temperature, even as the sample remains so far from the Curie point or any other transition temperature? We consider the temperature dependence of the different interactions responsible for the appearance of magnetic domains. The shape anisotropy tracks the average magnetization decrease with temperature, so it does not change much in the 300–500 K range. The magnetostriction decreases quite slowly above room temperature^22^. However the magnetocrystalline anisotropy decreases by a factor of 3 in that range^23^. As the magnetocrystalline anisotropy is the parameter that shows a larger variation, we consider how it might be responsible for the observed changes.

To check this idea, we perform micromagnetic simulations. Such simulations, incorporating shape and magnetocrystalline anisotropy, have been previously performed on magnetite cubes with (001) sides and sizes as large as 4 *μ*m^24^. These simulations show that the magnetization close to the surface is aligned within each surface plane, and thus away from the bulk easy axis directions. Furthermore curved domain walls were observed on the surface while further into bulk they turned into straight ones. Motivated by this reasonable initial agreement with some features of our observations, we have performed further micromagnetic simulations. To mimic our experimental situation near the surface region of a macroscopic single crystal, the simulations have been performed in a slab with two different regions. The lower one has the magnetization fixed through all the region to a bulk [111] direction. The upper region is left free to change the magnetization orientation in three dimensions.

**Figure 4:**
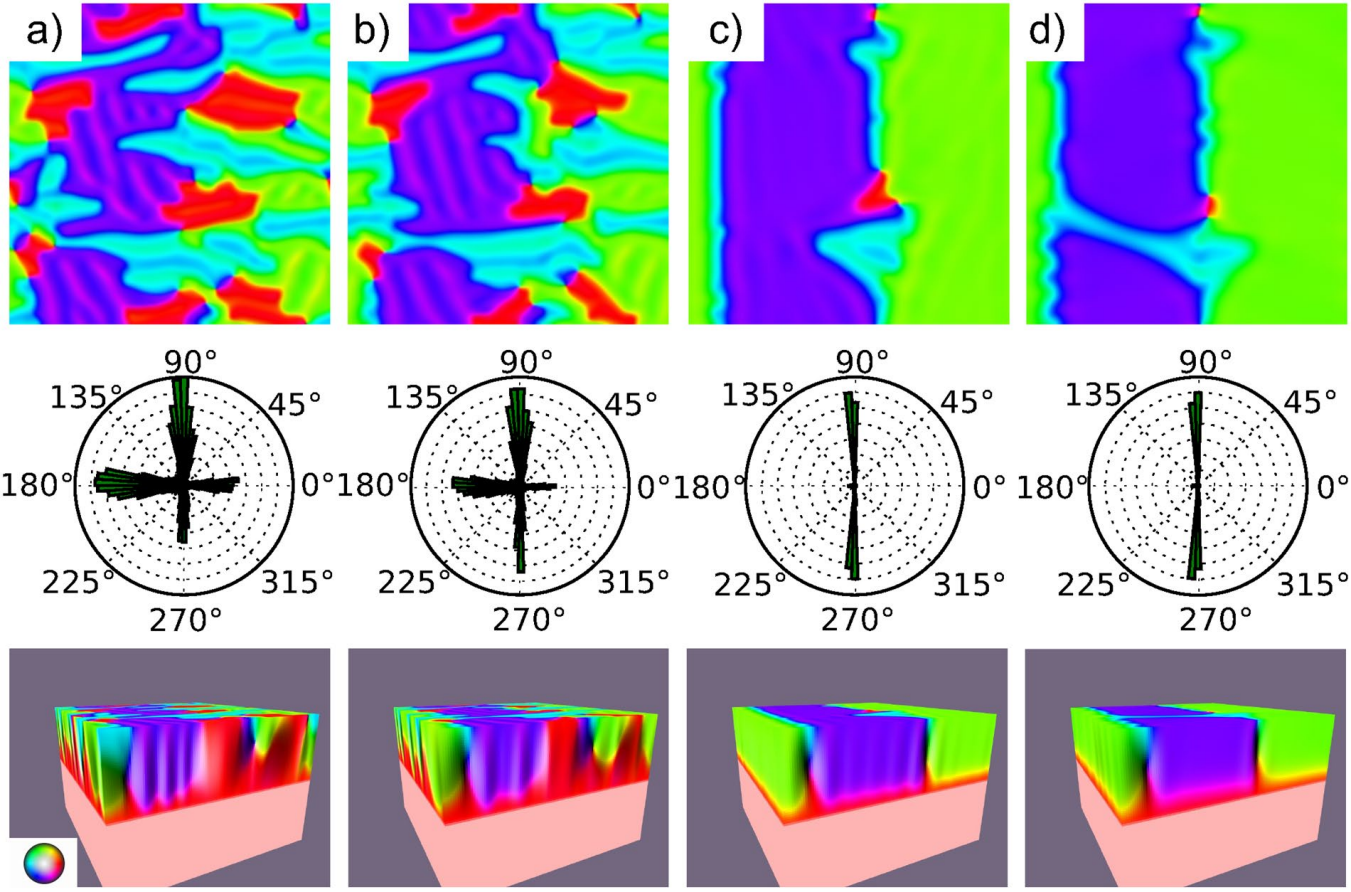
The top row shows a view of the surface magnetization from a micromagnetic simulation of a relaxed free slab of 840 nm on top of another 840 nm of a slab with a fixed [111] magnetization, with a lateral size of 10.24 *μ*m. The middle row shows the histogram of the in-plane distribution of the magnetization. The bottom row shows a side view of the slab using the MuView software^45^. *K*_1_ is set to −1.25·10^4^ in (**a**), −0.94·10^4^ in (**b**), −0.62·10^4^ in (**c**) and −0.3·10^4^ Jm^−3^ in (**d**) from left to right respectively. The colourmap employed to the top and side views is the same as in Fig. 1.

We have started with the same material parameters employed in ref.^24^, corresponding to magnetite at room temperature. With an initial random magnetic configuration, the torque is minimized using a Bogacki-Shampine solver with the MuMax3 code^25^. For free layers thinner than 80 nm, the magnetization turns uniformly from the bulk direction to the projection of the bulk direction into the surface plane (i.e., from the [111] to the [110] direction) just above the fixed magnetization slab, giving rise to a uniform surface magnetization in the rest of the upper slab. When the thickness of the free layer is increased, the upper surface of the slab breaks into several areas in which the magnetization is mostly along a given direction (Fig. 4a). The same orientation of the crystallographic axes and colour map are used as in the experiment (shown in Fig. 1d). A histogram of the magnetization from the top of the slab (Fig. 4a, middle) shows that the average orientation in each domain is, as expected, along an in-plane [110] direction, albeit with a significant spread of up to 15°. The observed in-plane canting in bands is reminiscent of the experimental data (see for example the upper left corner of Fig. 1b), as are the wavy domain walls between areas with the average magnetization in different 〈110〉 directions. A side view of the simulation slab (Fig. 4a, bottom) shows that the out-of-plane component of the magnetization reaches a maximum at the middle of the free slab, decreasing towards the surface.

Then the magnetization is reset to the initial random one, the magnetocrystalline anisotropy is decreased and the total torque is minimized again. This is repeated from the initial value of −1.25·10^4^ to −0.3·10^4^ Jm^−3^ (the alternative procedure of starting at each step from the previous configuration gives only minor changes in the patterns). The bands are reduced until they mostly disappear, as shown in the sequence of images in Fig. 4b–d. At the last step, corresponding to the magnetocrystalline anisotropy of −0.3·10^4^ Jm^−3^, the domains have a uniform magnetization, and the domain walls between them are quite straight. The reduction of the bands within each domain is nicely shown by the histograms, and like in the experimental case, their periodicity does not change much. Thus, varying the magnetocrystalline anisotropy in the range of values expected for magnetite in the measured experimental range presented in Fig. 2 produces changes in the domain patterns that resemble the evolution detected in the experiment. However, the overall distribution of domains is not similar. This can be attributed first to the limited size of our slab compared with the experimental situation. Another possible origin of the structures that we do not see in the simulation, the rombohedral domains, might also be related to the omission of magnetostriction effects in the simulations.

In any case, the suggestion that the magnetocrystalline anisotropy is responsible for the observed evolution is supported by the micromagnetic simulations. An additional test can be devised by noting the non-monotonic temperature dependence of *K*_1_. As mentioned before, *K*_1_ decreases in magnitude not only when heating above room temperature, but also when cooling down below 210 K. Thus, one might expect that the same evolution of magnetic domains on the surface of magnetite (001) should be observed upon cooling from 200 to 150 K. Of course, once the spin-reorientation transition is reached, the easy-axes will change and the behavior of the domains will differ (strongly) from the above room-temperature evolution.

**Figure 5:**
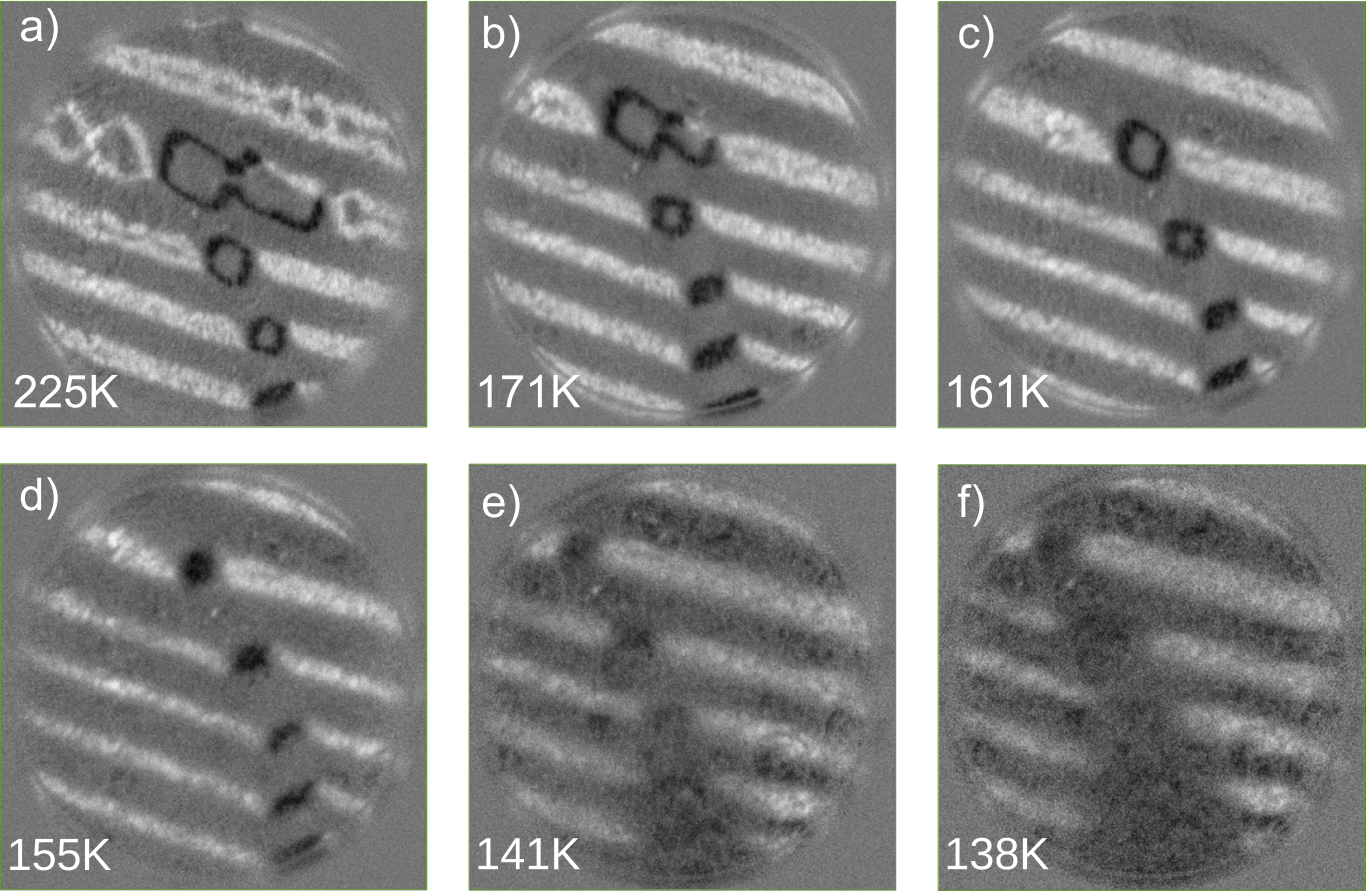
(**a**–**f**) SPLEEM asymmetry images showing the magnetization component along the x-axis, selected from a sequence of images saved while cooling from 225 to 138 K. The field of view is 12 *μ*m.

A sequence of images cooling down at the same area of the crystal that was followed above room temperature in Fig. 3 is presented in Fig. 5. The initial configuration is again composed of stripes and rombohedral domains. Upon cooling the rombohedral domains collapse into lines, a behavior that is again reversible upon warming up the temperature (not shown). We note, however, that the second stage of changes upon heating where the striped domains disappear gradually is not observed when cooling below room temperature. Instead, if the temperature is lowered enough the spin reorientation transition is encountered, at which point most surface magnetic domains unpin and change their easy axes^15^. We further note that unlike *K*_1_, neither the saturation magnetization nor the magnetostriction^5^ constants change appreciably below room temperature until the Verwey transition, ruling out that they are responsible for the observed changes.

Now we turn to the origin of the reproducibility of the magnetic domains before and after annealing to the Curie temperature. We first note that with spin-polarized electron microscopy we are looking at domains at the near surface region^21^. This is consistent with some of the features of the micromagnetic simulations. It can be argued that the large scale configuration of the magnetic domains deep into the crystal is not changing as much as in the near surface region. Then when the sample is cooled again, the bulk deep domains act as a template to which the surface domains have to adapt.

**Figure 6:**
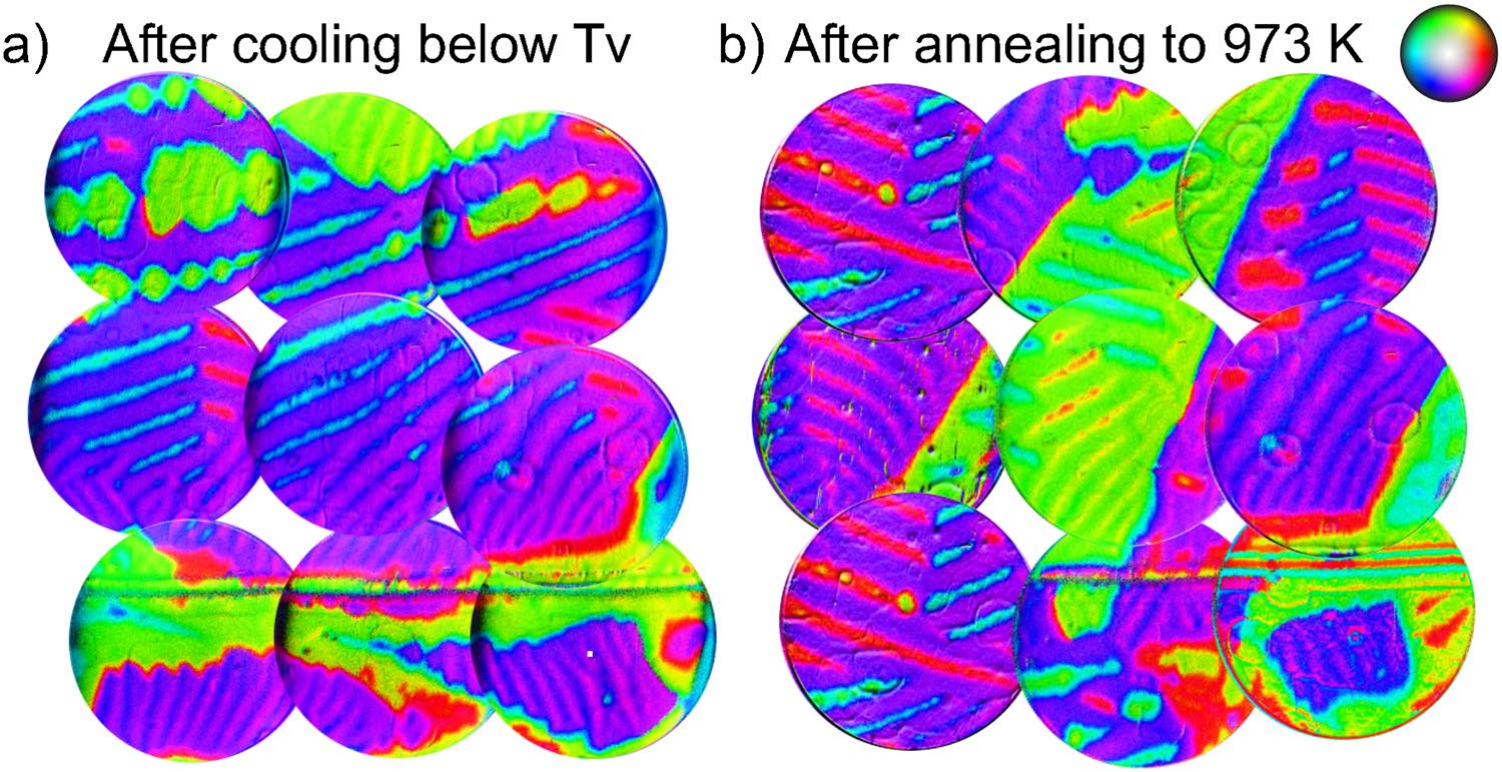
(**a**) Vector magnetization map of the same area shown in Fig. 1, acquired at room temperature after cooling down below the Verwey transition. (**b**) Vector magnetization of the same area, after annealing the crystal to 1000 K and cooling back to room temperature. As in the first figure, the area covered is 30 × 30 *μ*m^2^ (each individual image has a field of view of 12 *μ*m). The x-axis and y-axis directions correspond to [110] and $[\bar{1}\mathrm{10]}$ respectively.

Such a memory effect also occurs in samples cooled below the Verwey transition, which motivates the so-called low-temperature demagnetization (LTD) method. This procedure^14,26^, used in paleomagnetism for isolating single-domain-like remanence in multidomain magnetite, consists of cooling the magnetite sample through the Verwey transition and back to room temperature in zero field. This has been found to remove a large part of the multidomain remanence due to loosely pinned domain walls, as described by Ö. Özdemir *et al*.^27–29^. Microscopic observation of the magnetization domains of crystals subject to LTD have been performed by Kasama *et al*.^13^. In this case, the effect arises from the interplay of stress and magnetization across the Verwey transition. The final result is a magnetic structure that “is similar to that measured initially at 300 K […] but not exactly the same”^13^. We have performed the LTD procedure on our crystal. The result is shown in Fig. 6a. As the comparison with Fig. 1b,c attests, only minor changes of the magnetization pattern are detected.

However, the large scale memory effect we observe at high temperature must have a different origin, as the sample has been heated above the Curie temperature. It is know that defects can pin down the magnetic domains^27^, but it is difficult to understand how defects can fix the domain orientation. Instead we suggest that this high-temperature memory effect results from hematite inclusions. Such inclusions are a common occurrence in natural magnetite crystals as hematite is the stable oxide form in air, and their role has long been debated when discussing the transformation from magnetite to hematite^30,31^. More directly related to our sample, the well-established method to obtain a reproducible magnetite surface in ultra-high vacuum studies promotes the growth of hematite inclusions^18,32^. The literature recipe used to obtain a reproducible (√2 × √2)R45° reconstructed (100) surface^33^ involves repeated cycles of consecutive ion sputtering (to remove contaminants), and annealing steps in molecular oxygen (to order the surface and recover the preferentially sputtered oxygen). Such method produces hematite inclusions due to the local oxidation of magnetite to hematite, freeing iron that migrates to the surface to give rise to local magnetite growth^32^. Hematite is weakly ferromagnetic between the Morin transition temperature at 260 K and 956 K where it becomes paramagnetic^3^. The weak ferromagnetism is due to partial canting in the antiferromagnetic ordering. In similar crystals we have observed hematite inclusions with irregular shapes and sizes in the range of several micrometers. The large size of these inclusions is expected to suppress super-paramagnetic loss of remanence up to very close to the hematite transition temperature of 956 K. In essence, while the magnetite matrix was in a paramagnetic state during the 873 K annealing condition, the hematite inclusions remained ferromagnetic. As a result, the inclusions template the nucleation of the bulk magnetic domains of magnetite through the long range dipolar interaction upon cooling below the magnetite Curie temperature. These bulk domains in turn would template the evolution of the surface domains, following the change of magnetocrystalline anisotropy. This hypothesis suggests a simple test: annealing the crystal above the Néel temperature of hematite should erase the memory effect. In agreement with this, in Fig. 6b we shown how heating to 973 K effectively induces changes on the overall magnetic domain distribution which no longer resemble the original pattern.

It would be interesting to study the magnetic domains in magnetite (001) films as a function of film thickness. In fact, magnetite films have been the subject of a concerted growth and characterization effort in order to employ them for spintronic applications^34^. However, the magnetic properties of such films are strongly affected by growth defects such as antiphase domain boundaries^35,36^ making it difficult to observe the kind of effects we are reporting here. Given the deleterious effects of such defects in many magnetite properties^37^, the current efforts to grow more perfect films will hopefully provide samples where such studies can be attempted.

## Conclusions

By means of spin-polarized low-energy electron microscopy we have observed the magnetic microstructure on the surface of magnetite (001) and its evolution with temperature. A strong memory effect is detected, by which the surface remembers even small details of the magnetization upon annealing to the Curie temperature. Furthermore, the detailed evolution of the surface microstructure is found to be reversible and very similar in the temperature ranges of 300–500 K and 200–150 K, suggesting that the origin of the observed evolution is related to the temperature dependent changes of the first order magnetocrystalline anisotropy. Some of the observed changes can be reproduced by micromagnetic simulations lending further support to this hypothesis. The origin of the memory effect is suggested to be the presence of large hematite inclusions that remain magnetically ordered above the magnetite Curie temperature.

## Methods

### Experimental Methods

The experiments have been performed in a low-energy electron microscope (LEEM)^21,38^ equipped with an spin-polarized electron source and a spin manipulator to adjust the spin direction of the electron beam with respect to the sample surface^16^, which has been described previously^39–43^. Magnetic contrast is detected along a specific spatial direction by computing pixel-by-pixel asymmetry images from LEEM images acquired with the spin-polarization of the illuminating electron beam along opposite directions. To determine the magnetization vector, SPLEEM asymmetry images are acquired with the beam spin polarization aligned in orthogonal directions. Then the pixel intensity of the individual images corresponds to the Cartesian components of the magnetization vector. To represent it, a colour-intensity map is employed where in-plane orientation is indicated by colour and the out-of-plane angle by brightness, as shown in Fig. 1. The integration time required per LEEM frame is typically 1 second, and to change the beam spin direction takes a fraction of a second. Commonly the specular beam is used for imaging, in particular for all the grid maps, with a start voltage (i.e. the potential difference between the electron gun and the sample) of 8 V. In the temperature sequences, a first order diffracted beam has been employed as we have observed that it provides a larger image asymmetry, with a start voltage of 9 V.

In addition to the SPLEEM chamber an auxiliary ultra-high vacuum chamber accessed through a transfer rod houses a cylindrical mirror analyzer for Auger electron spectroscopy and a low-energy electron diffractometer.

The sample is a (001)-oriented single-crystal of natural origin^44^. The sample was cleaned after introduction into the SPLEEM system by a few cycles of 10 minutes sputtering with Ar ions at 1 keV followed by annealing to 870 K in 10^−6^ Torr of O_2_ for tens of minutes. After four cycles, the Auger spectra showed only iron and oxygen, and the low-energy electron diffraction pattern showed a sharp (√2 × √2)*R*45° reconstruction^33^. The sample was heated in ultra-high vacuum by electron bombardment from a tungsten filament, and cooled by circulating cooled nitrogen gas through the sample holder stage. Below room temperature the sample temperature was estimated by means of a Pt1000 thermometer in contact with the sample holder. At temperatures above 500 K the sample temperature was measured with an infrared pyrometer.

### Simulation Methods

The micromagnetic simulations were performed with the MuMax3 software^25^ using a low-end graphic GPU (2 Gb GeForce GTX760). The maximum simulation size was 256 × 256 × 165 cells. To simulate a 10 *μ*-wide slab, a rectangular unit cell of 40 × 40 × 10 nm was employed. Following W. Williams^24^, in such case the simulation should be considered qualitative. However the coarse large scale structure would still be representative of the expected domain distribution. In order to simulate the situation at the surface of a near infinite crystal, the simulations were performed in a slab divided into two regions along the z-direction: the lower half with a fixed (bulk-like) [111] magnetization orientation, and the upper half free to relax the magnetization in three dimensions. The initial configuration was a random one for the upper half of the slab, and the total torque was then minimized. Up to 10 replicas of the slab where used to simulate XY boundary conditions. The material constants employed for the saturation magnetization and exchange stiffness were *M*_*s*_ = 4.8 × 10^5^ A/m and *A*_*ex*_ = 2.64 × 10^−11^ J/m respectively^24^. The values for the first order magnetocrystalline cubic anysotropy were changed between −1.25 × 10^4^ and −0.30 × 10^4^ J/m^3^, starting at each time from the same initial random configuration.

### Data availability

The datasets generated during and/or analysed during the current study are available from the corresponding author on reasonable request.
